# Intracerebroventricular injections of dronabinol, a cannabinoid receptor agonist, does not attenuate serotonin-induced apnea in Sprague-Dawley rats

**DOI:** 10.1186/s12952-016-0052-1

**Published:** 2016-05-02

**Authors:** Michael W. Calik, David W. Carley

**Affiliations:** Center for Narcolepsy, Sleep and Health Research, University of Illinois at Chicago, 845 South Damen Avenue (M/C 802), Chicago, IL 60612 USA; Department of Biobehavioral Health Science, University of Illinois at Chicago, 845 South Damen Avenue (M/C 802), Chicago, IL 60612 USA; Department of Medicine, University of Illinois at Chicago, 1853 West Polk Street (M/C 784), Chicago, IL 60612 USA

**Keywords:** Dronabinol, Cannabinoids, Obstructive sleep apnea, Reflex apnea, Serotonin, Intracerebroventricular injection

## Abstract

**Background:**

Evidence suggests that vagal nerve activity may play a role in sleep apnea induction. In anesthetized rats, dronabinol, a cannabinoid (CB) receptor agonist, injected into the nodose ganglia attenuates reflex apnea and increases genioglossus activity, and reflex apnea attenuation is blocked by systemic pre-treatment with cannabinoid type 1 and/or type 2 receptor antagonists. However, it is unclear whether dronabinol has similar effects in the central nervous system; CB receptors are widely distributed in the brain, especially on neuronal circuitry important for respiration and upper airway activation. Here, we examine the effects of intracerebroventricular (ICV) injection of dronabinol on serotonin (5-HT)-induced apnea.

**Methods:**

Adult male Sprague-Dawley rats were anesthetized and instrumented with bilateral electrodes to monitor genioglossi EMG and with a piezoelectric strain gauge to monitor respiratory pattern. Serotonin was intravenously infused into a femoral vein to induce reflex apnea. After baseline recordings, rats were placed in a stereotaxic apparatus. A unilateral osteotomy was made to allow access for injection to the right lateral ventricle, and the dura were carefully removed. Dronabinol (100, 10, 1, or 0.1 μg/3 μl DMSO) or control (3 μl DMSO) was injected into the right lateral ventricle and 5-HT infusion was repeated. Data (mean ± SEM) were analyzed using a mixed model analysis with a repeated/fixed measure.

**Results:**

There was no main effect in 5-HT-induced apnea or breath duration, or in breath instability, between ICV dronabinol injected and ICV vehicle control injected groups. Moreover, there was no main effect in phasic or tonic genioglossus activity between ICV dronabinol injected and ICV vehicle control injected groups.

**Conclusion:**

Our data show that ICV injection of dronabinol did not decrease 5-HT-induced apneas, and did not increase genioglossus activity. This in contrast to published results of dronabinol’s effect on apnea via the vagus nerve. Our results suggest that the effects of dronabinol on reflex apneas are peripherally mediated via suppression of vagal nerve activity.

## Background

Obstructive sleep apnea (OSA), characterized by a cessation of breathing produced by a narrowed or collapsed upper airway, represents a significant public health risk [1, 2]. Current OSA prevalence estimates indicate that 14 % of American men and 5 % of American women suffer from OSA, and that those estimates are rising [1]. More importantly, there are strong associations between OSA and other diseases, such as type 2 diabetes, hypertension, stroke, and coronary heart disease [3–5]. The “gold standard” of OSA treatment is to splint open the upper airway via continuous positive air pressure; however, treatment tolerance is low and patients do not adhere to treatment despite obvious health benefits [2]. Safe and effective pharmacological treatments for OSA remain to be identified, and such efforts have been limited by incomplete knowledge of the central and peripheral neural mechanisms controlling respiration during sleep [2, 6]. Recently, activating the inhibitory G_i/o_-associated cannabinoid (CB) receptors have been proposed as novel pharmacological intervention to treat OSA [7, 8].

The endocannabinoid system, associated with cannabinoid type 1 (CB_1_) and cannabinoid type 2 (CB_2_) receptors located on peripheral nerves and central neurons, can be targeted therapeutically to modify disease states [9, 10]. Dronabinol, a synthetic version of Δ9-THC, is a FDA-approved CB_1_ and CB_2_ receptor agonist used to suppress chemotherapy-induced nausea and stimulate appetite in AIDS patients [11]. Dronabinol, when administered to patients with OSA [12], or to rats chronically-instrumented to measure respiration during sleep [13], decreased apneas. The mechanism of dronabinol’s effect in decreasing apnea propensity appeared to be, in part, due to the activation of both CB_1_ and CB_2_ receptors located on nodose ganglia of the vagus nerves [14, 15], which transmit vital information from the lungs to the brainstem, contributing to reflex responses regulating: tidal volume, respiratory frequency, augmented breaths and bronchoconstriction [16, 17]. In a well-established model of vagally-mediated reflex apnea [18], dronabinol injected into nodose ganglia attenuated apneas [15]. Antagonism of CB_1_, CB_2_, or both reversed dronabinol’s attenuation of apneas [14]. More importantly, dronabinol also increased phasic upper airway activity via activation of CB receptors at the nodose ganglia [14, 15]. Though recent evidence confirms the role the vagus nerve plays in apnea propensity [14, 15, 19–21], less is known about the role that central CB receptors have in apnea induction or suppression. Dronabinol is highly lipophilic and readily crosses the blood-brain barrier into the central nervous system [22], where CB receptors are widely distributed [23, 24] [10], including brain areas vital to respiratory control [23, 25–28]. Complicating the issue further is that activating central CB receptors can inhibit evoked release of excitatory or inhibitory neurotransmitters [29], thereby inhibiting or disinhibiting neuronal activity [9, 30]. It is unknown if central versus peripheral administration of a CB agonist would have similar or dissimilar effects on respiration. Therefore, it is important to understand dronabinol’s global effects on the central nervous system without activating CB receptors in the peripheral nervous system.

Here, we hypothesized that global central administration via intracerebroventricular (ICV) injection of dronabinol would attenuate reflex apneas and increase upper airway activity.

## Methods

### Animals

Thirty adult male Sprague-Dawley rats (275–300 g) were purchased from Harlan Laboratories (Indianapolis, IN, USA), housed in duplicate, maintained on a 12:12 light:dark cycle at 22 ± 0.5 °C, and allowed *ad libitum* access to food and water. All animal procedures and protocols were approved by the Institutional Animal Care and Use Committee of the University of Illinois at Chicago (Protocol no.: 11–217/14–159).

### Acute ICV injection experiment paradigm

Rats (*N* = 30) were anesthetized (IP ketamine:xylazine 100:10 mg/kg; IP redosing 100:5 mg/kg; surgical plane of anesthesia was monitored by toe pinch) and instrumented with bilateral electrodes to monitor genioglossus EMG (EMGgg; 1 mm lateral to the midline) and with a piezoelectric strain gauge to monitor respiratory pattern. The femoral vein was cannulated for 5-HT (12.5 μg/kg; MP Biomedicals, Solon, OH, USA) in PBS (pH 7.4; 0.35 ml/kg) infusions via an infusion pump (63 ml/h; KD Scientific, Holliston, MA, USA) to induce reflex apneas (repeated a minimum of two times). After baseline recordings (*N* = 30), the head of the rat was mounted in a stereotaxic frame, and dorsal craniotomy was performed to allow for right ICV injections of dronabinol (10 mg capsules, *Marinol*, Abbvie Inc., North Chicago, IL, USA) at various concentrations (100, 10, 1, or 0.1 μg/3 μl DMSO; *N* = 6 for each concentration) or vehicle control (3 μl DMSO; *N* = 6) using a 28 gauge needle. Dronabinol ICV doses were chosen based on other physiological effects of various Δ9-THC ICV doses [31–34]. After ICV injections over a minimum 3 min period, 5-HT infusions were performed again to induce reflex apneas (repeated a minimum of two times).

### Data recording and processing

Data recording and processing have been described before [14, 15]. Briefly, during EMGgg and respiratory data acquisition, signals were amplified and band-passed filtered (10–240 Hz and 1–10 Hz, respectively; CyberAmp 380, Axon Instruments, Sunnyvale, CA, USA), digitized at 500 Hz (Data Acquisition Subsystems, DataWave Technologies, Loveland, CO, USA), and recorded and saved using SciWorks Experimenter software (DataWave Technologies, Loveland, CO, USA). After recording, EMGgg data were rectified and smoothed (time constant of 100 ms) using Spike2 software (Cambridge Electronic Design, Cambridge, England). Tonic EMGgg was defined as the nadir of smoothed expiratory genioglossus activity. Phasic EMGgg was defined as the peak of smoothed inspiratory genioglossus activity minus tonic EMGgg. EMGgg signals after ICV injections were normalized by dividing by EMGgg signals recorded before ICV injections, and are reported as arbitrary units (a.u.). Breath durations, and phasic and tonic EMGgg amplitudes were averaged from 5 previous breaths before IV 5-HT infusion; this was repeated a minimum of two times and averaged. Apnea durations were defined as the average of the longest breath durations lasting at least 2.5 seconds [13] within 30 seconds following IV 5-HT infusion. For measurement of respiratory instability [35], coefficient of variation was calculated for 30 breath durations before and after injection of 5-HT.

### Statistical analysis

Data (mean ± SEM) were analyzed using IBM SPSS Statistics 22 (New York, NY, USA) mixed model analysis with a repeated/fixed measure (ICV treatment) or two-way repeated/fixed measure (time × ICV treatment) followed by post hoc multiple comparison tests with Sidak’s correction if there was a significant main effect. Repeated covariance structure was chosen according to the best-fit Schwarz’s Bayesian information criterion. Statistical significance was set at *p* < 0.05.

## Results

**Fig. 1:**
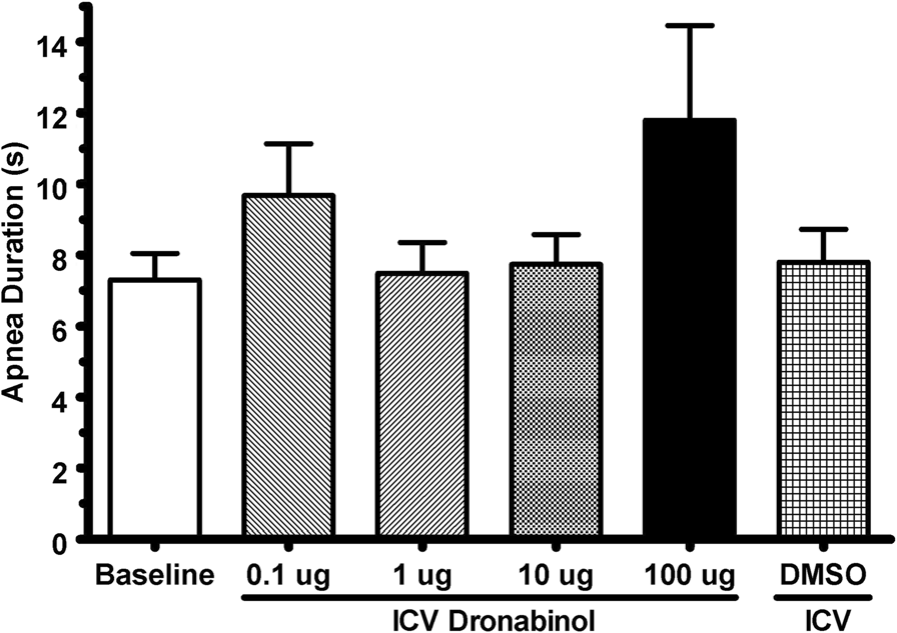
Apnea duration quantified from acute 5-HT-induced apnea experiments before (baseline; *N* = 30) and after ICV injections of various concentrations of dronabinol (100, 10, 1 or 0.1 μg; *N* = 6 for each dose) or vehicle (DMSO; *N* = 6). ICV injections of dronabinol at any concentration did not significantly (*p* = 0.19) attenuate reflex apneas. Data (mean ± SEM) were analyzed using mixed model analysis with a repeated/fixed measure (ICV treatment)

**Fig. 2:**
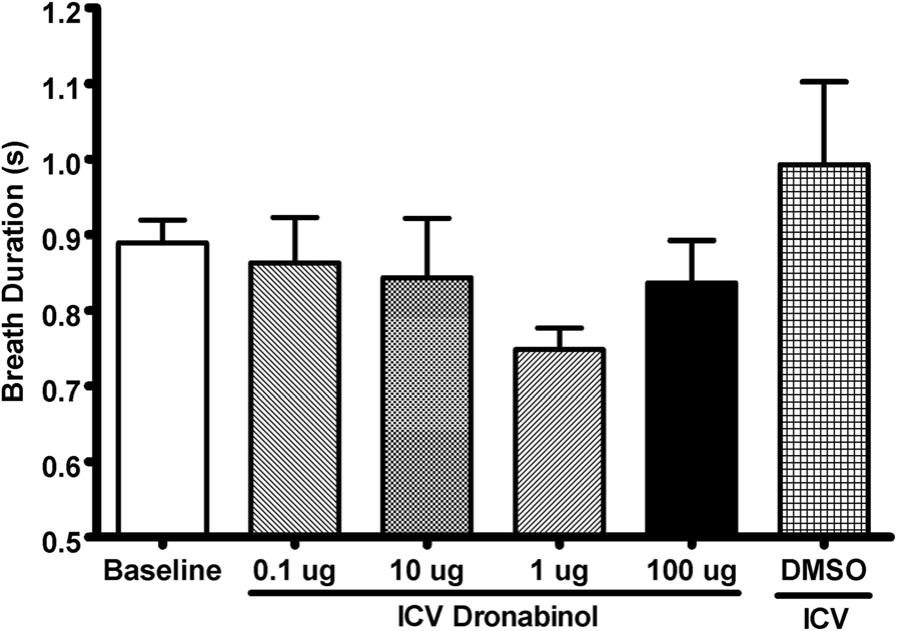
Breath duration quantified prior to 5-HT infusion before (baseline; *N* = 30) and after ICV injections of various concentrations of dronabinol (100, 10, 1 or 0.1 μg; *N* = 6 for each dose) or vehicle (DMSO; *N* = 6). There were no significantly (*p* = 0.12) differences in breath duration in the treatment groups. Data (mean ± SEM) were analyzed using mixed model analysis with a repeated/fixed measure (ICV treatment)

**Fig. 3:**
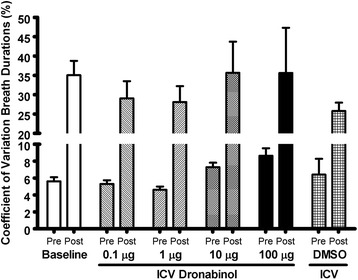
Coefficient of 30 breath durations (%) quantified prior (pre) to and after (post) 5-HT infusion before (baseline) and after ICV injections of various concentrations of dronabinol (100, 10, 1 or 0.1 μg; *N* = 6 for each dose) or vehicle (DMSO; *N* = 6). There were no significant differences in ICV treatment (*P* = 0.45) or interaction between time and ICV treatment (*p* = 0.46). However, there was a main effect of time (*p* < 0.01), with post hoc analysis showing a significant increase in respiratory instability after 5-HT infusion (*p* < 0.01). Data (mean ± SEM) were analyzed using mixed model analysis with a two-way repeated/fixed measure (time × ICV treatment)

**Fig. 4:**
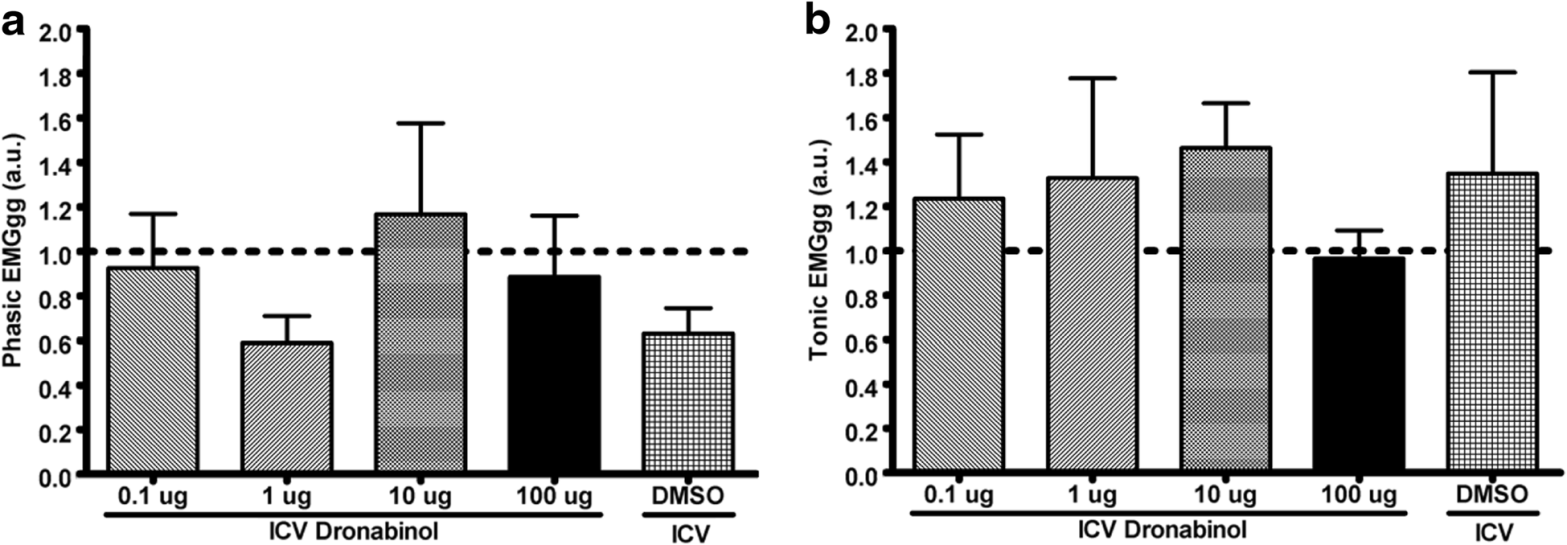
Phasic (**a**) and tonic (**b**) genioglossus electrogram amplitude (arbitrary units, a.u.; dotted line corresponds to baseline EMGgg) quantified after ICV injections of various concentrations of dronabinol (100, 10, 1 or 0.1 μg; *N* = 6 for each dose) or vehicle (DMSO; *N* = 6). There were no significant differences in phasic (*p* = 0.48) or tonic (*p* = 0.37) EMGgg in the treatment groups. Data (mean ± SEM) were analyzed using mixed model analysis with a repeated/fixed measure (ICV treatment)

Reflex apneas induced via IV infusion of 5-HT were conducted in rats before and after ICV injections of various concentrations of dronabinol (Fig. 1). Also, breath duration, coefficient of breath durations, and phasic and tonic genioglossus activity were quantified before and after ICV injections of various concentrations of dronabinol (Figs. 2, 3, and 4, respectively).

At baseline (before ICV injections), IV 5-HT infusion produced reflex apneas lasting 7.29 ± 0.75 seconds (*N* = 30). ICV injections of 100 μg (11.80 ± 2.66 seconds, *N* = 6), 10 μg (7.73 ± 0.85 seconds, *N* = 6), 1 μg (7.48 ± 0.87 seconds, *N* = 6), or 0.1 μg (9.67 ± 1.46 seconds, *N* = 6) of dronabinol, or injection of vehicle (DMSO; 7.79 ± 0.93 seconds, *N* = 6) did not significantly (*F*_5, 35.6_ = 1.90, *p* = 0.12 for main effect of “treatment”) alter apnea durations compared to baseline (Fig. 1).

Breath durations were averaged from 5 breaths prior to 5-HT-induced apneas. Average breath duration before ICV injections was 0.89 ± 0.03 seconds (*N* = 30), and was not significantly (*F*_5, 32.7_ = 1.89, *p* = 0.12) altered by 100 μg (0.84 ± 0.06 seconds, *N* = 6), 10 μg (0.84 ± 0.08 seconds, *N* = 6), 1 μg (0.75 ± 0.03 seconds, *N* = 6), or 0.1 μg (0.86 ± 0.06 seconds, *N* = 6) ICV injection of dronabinol, or ICV injection of vehicle (DMSO; 0.99 ± 0.11 seconds, *N* = 6; Fig. 2). Coefficient of variation of 30 breath durations before (pre) and after (post) 5-HT infusion was quantified as a measurement of respiratory instability (Fig. 3) [35]. There was no ICV treatment main effect (*F*_5, 11.0_ = 1.03, *p* = 0.45) or interaction between ICV treatment and time (*F*_5, 11.0_ = 1.01, *p* = 0.46). There was a main effect of time (*F*_1, 14.5_ = 87.7, *p* < 0.01); breathing was more unstable following 5-HT infusion compared to before infusion (*p* < 0.01; Fig. 3).

Genioglossus activity was measured at the start of inspiration (Phasic EMGgg) and at the end of expiration (tonic EMGgg) prior to reflex apneas (Fig. 4). ICV Injections of 100 μg (0.89 ± 0.28 a.u., *N* = 6), 10 μg (1.17 ± 0.41 a.u., *N* = 6), 1 μg (0.59 ± 0.12 a.u., *N* = 6), or 0.1 μg (0.93 ± 0.24 a.u., *N* = 6) of dronabinol, or injection of vehicle (DMSO; 0.63 ± 0.11 a.u., *N* = 6) did not significantly (*F*_4, 9.50_ = 0.94, *p* = 0.48 for main effect of “treatment”) alter phasic EMGgg (Fig. 4a). Similarly, ICV injections of 100 μg (0.97 ± 0.13 a.u., *N* = 6), 10 μg (1.46 ± 0.20 a.u., *N* = 6), 1 μg (1.33 ± 0.45 a.u., *N* = 6), or 0.1 μg (1.23 ± 0.29 a.u., *N* = 6) of dronabinol, or injection of vehicle (DMSO; 1.35 ± 0.46 a.u., *N* = 6) did not significantly (*F*_4, 7.96_ = 1.22, *p* = 0.37 for main effect of “treatment”) alter tonic EMGgg (Fig. 4b).

## Discussion

With the increasing prevalence [1], comorbidity with other diseases [3–5], and a lack of tolerable and effective treatment options [2, 6], OSA is a significant health problem. An impediment to effective treatments of OSA is insufficient knowledge of peripheral and central neural mechanisms of respiratory control, especially during sleep. Though we have shown previously that modulation of vagal afferents via activation of CB receptors located on nodose ganglion neurons can attenuate reflex apneas [14, 15], it was uncertain what role central CB receptors play in respiratory pattern control. Here we show that ICV injection of dronabinol, a non-specific agonist of CB_1_ and CB_2_ receptors, does not attenuate peripherally-induced reflex apneas.

Vagal afferent neurons, which relay important information about respiratory drive and upper airway muscle tone [16, 17, 36], provide input to the excitatory or inhibitory neurons located in nucleus of the solitary tract (NTS) [37, 38]. The NTS projects to the respiratory centers of the brainstem, including the hypoglossal nucleus (XII) and the rostral ventrolateral medulla (RVLM) [36, 39]. The NTS contains a variety of neuronal populations that fire at distinct points in the expiratory-inspiratory phases of breathing [40]. Reflex apneas are induced peripherally via the vagus nerve by pharmacologically activating excitatory receptors located on nodose ganglia, or by mechanically activating stretch receptors located in the lung [16, 41]. Reflex apneas are also induced “downstream” by activation of glutamatergic receptors in the NTS [42–45]. Conversely, vagally-induced apnea can be reversed by microinjection of a GABA agonist [46], or a glutamatergic NMDA antagonist [45], into the NTS. Vagal afferents also synapse in regions of the NTS that modulate sympathetic activity, with activation of vagal afferents not only inducing apnea, but decreasing heart rate and blood pressure [41]. This trifecta of responses, known as the Bezold-Jarisch reflex, is modulated by different regions of the NTS, and these regions, when activated or inhibited, induce variable responses in blood pressure, heart rate, and breathing [45–47]. The NTS contains both CB_1_ and CB_2_ receptors that, when activated, inhibit or promote NTS activity, and attenuate other vagally-mediated behaviors [26, 27, 48–51]. CB_1_ activation decreases glutamate release in the NTS [51], thus CB_1_ activation would have the effect of suppressing apneas. CB_1_ activation also disinhibits second order NTS neurons by pre-synaptically decreasing release of GABA [51–53], thus CB_1_ activation would have the opposite effect of potentiating apneas. It might be plausible that the dual effects of CB_1_ activation occurred, with the consequence of neither suppressing nor potentiating apneas.

Moreover, the XII, which modulates the phasic and tonic activation of the genioglossus muscle [36], also contains CB_1_ receptors [23, 25, 28, 54, 55], of which the physiologic role is unclear. CB_1_ activation in the XII is known to disinhibit XII by preventing release of glycine, an inhibitory neurotransmitter [25, 54]. In fact, a CB agonist microinjected in the XII activated the genioglossus in awake, but not sleeping, rats [55]. A recent report showed cardiorespiratory anomalies, including unstable breathing and apneas, in CB_1_ knockout mice [56]. We hypothesized that the activation of CB receptors located at crucial respiratory centers in the brainstem would modulate reflex apneas and increase genioglossus activity; however, we saw no effect of a centrally-administered CB agonist, dronabinol.

The present work focused on global activation of CB receptors located in the brain, which mimicked patients undergoing dronabinol treatment [12], and did not elucidate any specific local effects of the respiratory centers of the brainstem. Due to the variability of Bezold-Jarisch reflex responses to NTS activation or inhibition, and the location of CB receptors on both excitatory and inhibitory neurons of the NTS, it is possible that non-specific and global activation of central CB receptors via ICV injection of dronabinol led to multiple and potentially opposing responses, masking any specific local effects. Therefore no physiologic response was observed. For example, Padley et al. induced apnea by microinjecting a CB_1_ agonist centrally into the RVLM, which sends projections to inhibitory neurons located in the respiratory centers of the brainstem [39]. Also, injection of CBs peripherally in the nodose ganglia increased genioglossus activity, presumably through disinhibition of parasympathetic input into respiratory centers of the brain [15]. Carley et al. showed respiratory stability with intraperitoneal injections of dronabinol, which would involve both peripheral and central CB receptors [13]. Moreover, low and high doses of CBs are known to have biphasic effects [31, 57]; however, we saw no changes in apnea response at low or high doses of dronabinol. Since CB receptors are widely distributed in the brain, on both excitatory and inhibitory neurons, and are activated differentially by different concentrations of CBs, specific microinjections in the NTS or XII will need to be completed to see if CBs have any effect on breathing and genioglossus activity in rats.

## Conclusions

In conclusion, we show that ICV injections of dronabinol, a non-specific CB agonist, had no effect on 5-HT-induced reflex apnea, and had no effect on genioglossus activity. These results suggest that central CB receptors have a minimal or no effect on breathing when activated globally; however, there might be specific local effects of CBs due to the diverse population of neuronal inputs and outputs of the NTS. Previous work showing stabilization of breathing from CBs might be derived from activation of CB receptors located on peripheral nerves [13–15], suggesting that pharmacotherapies targeting only peripheral CB receptors for OSA treatment might be sufficient. Future work will concentrate on elucidating specific local effects of CBs on breathing in the NTS and XII.

## Abbreviations

5-HT: serotonin
a.u.: arbitrary units
CB: cannabinoid
CB_1_: cannabinoid type 1 receptor
CB_2_: cannabinoid type 2 receptor
DMSO: dimethyl sulfoxide
ICV: intracerebroventricular
NTS: nucleus of the solitary tract
OSA: obstructive sleep apnea
RVLM: rostral ventrolateral medulla
XII: hypoglossal nucleus
